# 23.4 Briefing

**DOI:** 10.1111/hex.13127

**Published:** 2020-09-16

**Authors:** Joseph W LeMaster

**Affiliations:** ^1^ Department of Family Medicine and Community Health School of Medicine University of Kansas Kansas City KS USA

Multi‐morbidity has recently come to renewed international attention due to its impact on disease susceptibility and outcomes for people infected with SARS‐Cov‐2, the agent responsible for COVID‐19. People with multiple long‐term conditions are usually those in the highest 5% of health‐care spenders, and their care incurs approximately 60% of all health‐care costs, in the United States at least.^1^ There, the top 10% of Medicare beneficiaries have health‐care costs over 6 times those without Medicare, and incur Medicare payments over six times those of fee‐for‐service patients.^2^, ^3^, ^4^ People with multiple long‐term conditions often feel misunderstood or ashamed and struggle with a sense of personal disintegration.^5^, ^6^, ^7^ Thus, patient involvement with these people is important if efforts are to be effective that aim to intervene in ways that change outcomes that matter to these patients.

A number of studies in this issue of HEX draw attention to the challenges faced by those with chronic or co‐morbid diseases. Van Schelven et al conducted a scoping literature review that included 23 studies from 2002‐2017 that described involvement of young people with chronic conditions. The authors noted that studies in which involvement was the focus more systematically described involvement and impact than studies that only included involvement as a method. While PPI positively impacted project design, recruitment, data collection and analysis and dissemination of project results, time and funding limitations hindered conduct of projects, especially among ‘hard to reach’ youth. Those most effective involved youth at the earliest stage of the project, during the development/refinement of study questions. Use of visual methods, group work, regular meetings, avoidance of conflicts that involved power dynamics and co‐design of research products was particularly effective; however, there was little evidence that the approach to PPI developed over the period that the study reviewed.^8^

While the benefits of public and patient involvement in research or clinical practice improvement have been widely documented, practical frameworks to carry out this process that link researchers, patients and industry/other stakeholders is still a gap. Feeny et al report how 350 persons with Parkinson's disease and their care partners, trained in the academic research process, developed such a framework in partnership with The Parkinson Foundation. The framework specifies how patients and caregivers interacted with researchers to develop a scope of work, establish guiding principles, optimize (from the patients’ point of view) the selection and training of participants and choice of project metrics, and monitor the conduct of the project and disseminate its findings. The partnership collaborated on 444 tasks across 237 unique projects. They describe process (quality) and outcome metrics agreed upon with research stakeholders that capture the significance of patient engagement practices to researchers. Feedback was provided to all parties in the research project and publicly discussed whether PPI was executed with success. This framework may serve as a model for other PPI networks to emulate, in order to standardize research practice to include and report on patient engagement.^9^

Using data from a qualitative study that investigated team‐based care for patients with inflammatory arthritis, Hartford et al asked ‘How do patients with inflammatory arthritis experience influence, authority, and control in their health‐care decision within their care network?’

They explored how people's identity, control and sense of agency developed in terms of their relationship with a multi‐disciplinary care team. They found that open communication with patients that acknowledged their expertise regarding their own treatment was a significant factor in their activation of their self‐care knowledge, skill and confidence. Power negotiation, however, was an integral component of patient‐clinician relationships. This influenced assertiveness in care decisions with clinicians: patients used different strategies over time to avoid conflict and jeopardizing their treatment, such as playing the role of a ‘good patient who does not challenge medical authority’. The authors write ‘Emphasizing patients' illness accounts as a pathway to foster ties through which power and identity can be co‐constructed may be critical if patients are to be agentic partners in their treatment decisions and gain control of their health‐care management’. This suggests the importance of re‐imagining organizational structures that support the agency of patients in treatment decision‐making and that make a place for patients’ lived experience alongside professional expertise, research and organizational or public policy.^10^

McCarron et al conducted patient and family interviews to better understand participants’ motivation for involvement in health‐care decision‐making. Initial involvement came when there was opportunity and desire to give input, but continued when participants believed their input was recognized, valued and meaningful, and that they had developed productive and mutually beneficial relationships. This was especially true if it took place within the health‐care system. Improvements in perceived/changed attitudes of clinicians towards patients and family members were noted as of particular importance. The importance of perks, for example paid travel to conferences to co‐present, represented one means of recognition for their contribution of which participants made note.^11^

Faletau et al followed traditional methods, conducting semi‐structured interviews with 12 Tongans with pre‐diabetes to understand their concept of risk of developing type 2 diabetes, finding that awareness of that risk was minimal but provoked fear (when they became aware), that they had diabetes and that communication from clinicians that was both clinically accurate and culturally appropriate facilitated appropriate behaviour change.^12^

Schmajuk and colleagues undertook six focus groups with 25 patients with rheumatoid arthritis with varying levels of limited English proficiency and three with 11 clinicians to co‐design a rheumatoid arthritis dashboard that would promote patient‐clinician communication about patient‐reported outcome data and patient self‐management. Patients thought the dashboard would be helpful as a valuable way to communicate with rheumatologists and to coordinate their care across specialties. Clinicians agreed but were skeptical that the dashboard would work as intended and might not import data accurately from their EMR so that it could be used effectively in their clinic workflows.^13^

Racine et al conducted a mixed methods study in which 16 people with diabetes and 15 health‐care providers attended either a patients‐only, providers‐only or mixed meeting (patients and providers) to explore participants’ experience of group decision‐making regarding planned implementation in practice of in an intervention to improve uptake of retinopathy screening. Participants in the combined group felt undervalued by those in the other stakeholder group, that is patients felt undervalued by providers and felt defensive about expressing their opinions. This made it difficult for them to stay on task and make progress. The authors concluded that it might be better to involve patients and providers separately when designing an implementation strategy.^14^

Davies and colleagues used a realist approach to better understand how training health coaches to support those living with long‐term conditions works best, for whom and under what circumstances. After interviewing twenty health‐care professionals and two coaches, the authors concluded that growth in coaches’ confidence required ‘outside the box’ discussion regarding barriers to implementation in practice, and how existing teamwork structures and processes may influence the relevance with which coaching trainees view their training, including use of peer support and ongoing reflection about the training process.^15^

Cheng et al conducted a qualitative study involving 18 homebound adults over 50 years old with various co‐morbidities including chronic pain, to learn how becoming homebound affects patients’ ability to gain access to health care, and what changes might be helpful. Participants often felt that their office‐based appointments were rushed, giving the impression that the health‐care system prioritizes efficiency over their (patients’) needs. They reported that they were ‘experts of their own bodies’ and that some doctors seemed to have preconceptions which led to poor understanding of their pain; or that attention to physical problems at times occurred at the expense of psychological assessment or support. From their perspective, effective care requires interpersonal communication skills.^16^

Given the escalating number of people with chronic disease worldwide, we strongly encourage continued efforts to involve these patients in research that informs and guides improvement of their health care. Patients are ready, willing and able to take part; moreover, not to include them in the design of studies that affect them will not optimize their participation and compromise the studies’ long‐term impact.
